# Relationship Between Depression and Medication Adherence Among Chronic Disease Patients in the Middle East

**DOI:** 10.7759/cureus.69418

**Published:** 2024-09-14

**Authors:** Abdulrahman O Alomar, Rakan H Khushaim, Shahad K Al-Ghanem, Abeer T Bin Jumaiah, Suhail M Albaqami, Lujain A Alleft, Eman A Abahussain

**Affiliations:** 1 College of Medicine, Imam Mohammad Ibn Saud Islamic University, Riyadh, SAU; 2 Psychiatry, College of Medicine, Imam Mohammad Ibn Saud Islamic University, Riyadh, SAU

**Keywords:** chronic diseases, chronic illness, depression, depression and medication adherence, medication adherence, medication compliance, treatment compliance

## Abstract

Background and aim

Medication adherence is a crucial factor in the management of chronic diseases. Depression is a common psychiatric disorder that has been correlated with poor adherence to medication. Adherence to medication in patients with chronic diseases is a well-known concern among the medical community, particularly when considering the adverse outcomes of adherence failure in such patients. Therefore, this study aims to investigate the relationship between depression and medication adherence among chronic disease patients in the Middle East.

Methodology

This cross-sectional study was conducted between January 2024 and April 2024 among patients with chronic diseases from the Middle East. The data were collected through convenient sampling using structured online-based questionnaires that included demographic information, type of chronic condition, medication adherence (measured by the Adherence to Refills and Medications Scale (ARMS)), and depression severity (measured by the Patient Health Questionnaire-9 (PHQ-9)). Statistical analyses were conducted to examine the associations between these variables.

Results

The study involved 492 participants with chronic diseases and found significant associations between demographic factors and medication adherence. Female participants were more likely to be non-adherent than males (p = 0.001), and younger participants aged 20-30 years exhibited higher non-adherence rates (46.7%) compared to older participants, 61 years and above (16.1%, p = 0.001). Non-working individuals showed higher non-adherence (45.4%) compared to retired individuals (21.5%, p = 0.003). Depression severity was inversely related to medication adherence; participants with severe depression were significantly more likely to be non-adherent (relative risk (RR) = 8.41, 95% confidence interval (CI): 4.26-16.57, p < 0.001). In addition, certain chronic conditions were more impactful on medication adherence and depression rates, such as patients with rheumatoid disease that had lower rates of none to minimal depression (8.6%) and higher rates of moderately severe - severe depression each (28.6%). Meanwhile, asthma patients showed notably lower adherence rates (41.0%) and higher levels of severe depression (24.6%).

Conclusion

The study highlights the intricate relationships between demographic factors, chronic conditions, medication adherence, and depression in patients with chronic diseases in the Middle East. Gender, age, occupational status, and marital status significantly influence adherence and depression levels. Depression is a significant factor associated with decreased levels of adherence among patients with chronic conditions. Regular screening to detect and manage depression among chronically ill patients is advised to help improve medication adherence.

## Introduction

The World Health Organization (WHO) defined chronic diseases as “diseases that have one or more of the following characteristics: they are permanent, leave residual disability, are caused by nonreversible pathological alteration, require special training of the patient for rehabilitation, or may be expected to require a long period of supervision, observation or care" [1]. Medication adherence is a crucial factor in the management of chronic diseases, as they tend to be of long duration and commonly require long-term therapies. The average adherence to long-term management for chronic diseases in developed countries is 50% according to the WHO. In addition, the estimated rate of medication non-adherence among patients with chronic diseases as reported by a review of studies from Middle Eastern countries ranged from 1.4% to 88% [1,2].

Some chronic conditions, such as diabetes mellitus, hypertension, and depression, have the highest rate of medication non-adherence [3]. Furthermore, poor adherence is a primary reason for suboptimal clinical benefit and can compromise the effectiveness of treatment resulting in poor outcomes [1].

Depression is a common psychiatric disorder that is characterized by abnormal and persistent low mood, accompanied by other symptoms including sleep disturbance, loss of appetite, suicidal thoughts, impaired concentration and attention, guilt, and pessimism [4-6]. Symptoms vary in severity, and the pattern of illness can range from an isolated and relatively mild episode, through recurrent episodes of moderate severity, to chronic and persistent severe illness [6].

Depression has been correlated with poor adherence to medication in previous literature conducted, as per a systematic review that was done on the factors affecting medication adherence in the older adult population found that patients with depression resulted in lower medication adherence [7]. Another study showed depression has been associated with an increase in non-adherence when compared to non-depressed patients in the treatment of chronic diseases [8].

In addition, a study in Jeddah, Saudi Arabia, revealed that there is an inverse association between depression and medication adherence among patients with chronic diseases [9]. Adherence to medication in patients with chronic diseases is a well-known concern among the medical community and with the growing evidence of depression having higher rates of poor adherence [10,11], identifying the association between medication adherence and depression is important particularly when considering the adverse outcomes of adherence failure on patients.

Therefore, this study aims to investigate the relationship between depression and medication adherence among patients with chronic diseases as few studies have been conducted in Saudi Arabia.

## Materials and methods

Study design

This cross-sectional study was conducted from January 2024 to April 2024 including patients with chronic diseases in the Middle East. Informed consent was obtained from all participants before administering the questionnaire, and the purpose of the study was clearly explained. The data were collected using an online-based questionnaire (see Appendix).

Study population

The study population included individuals aged 20 years and older, residing in Middle Eastern countries, and who have been diagnosed with chronic diseases. The respondents were recruited through convenient sampling, and the sample size was determined to be 385 participants, calculated using a sample size formula with a 95% confidence level and a 5% margin of error according to the Raosoft sample size calculator (Raosoft, Inc., Seattle, WA).

Study tool

Upon obtaining Institutional Review Board (IRB) approval, informed consent was obtained and the participants who agreed to take part in the study were asked to complete a digital questionnaire. This questionnaire was disseminated through various social media applications, including WhatsApp and Telegram group chats. The survey comprised 33 questions, integrating a validated Arabic version of the Patient Health Questionnaire (PHQ-9) for assessing depression and a validated Arabic version of the Adherence to Refills and Medications Scale (ARMS) for evaluating medication adherence. In addition, the questionnaire collected sociodemographic information such as age, sex, employment status, marital status, education, and income. The question types varied, encompassing multiple-choice questions, Likert scales, and yes/no questions. 

The study tools included the Arabic versions of the Adherence to Refills and Medications Scale (ARMS) and the Patient Health Questionnaire (PHQ-9). Inclusion criteria for participants were Middle Eastern residents, both male and female, aged 20 years or older, who had a chronic illness. Exclusion criteria included individuals under the age of 20, participants without a chronic illness, pregnant patients, and those who did not consent to participate in the study.

Data analysis

Data analysis was performed using Statistical Package for Social Studies (SPSS) version 26 (IBM Corp., Armonk, NY). Descriptive statistics were employed to outline the demographic characteristics of the patients, as well as the prevalence of depression and medication non-adherence among different chronic disease groups. Inferential statistics including the chi-square test, Student's t-test, and analysis of variance (ANOVA) test were used to investigate the association between depression and the risk of medication non-adherence in patients with chronic diseases. All statements were considered significant when the p-value was lower than 0.05.

Ethical consideration

Ethical approval for this study’s conduction and data collection were reviewed and approved by the Institutional Review Board (IRB) Committee of Al-Imam Muhammad Ibn Saud Islamic University (Project number: 647/2024).

## Results

Demographic characteristics of the participants

In this study, the total sample consisted of 492 individuals. Of these respondents, 163 (33.1%) were male and 329 (66.9%) were female. The age distribution showed that the largest group was between 20 and 30 years old, comprising 199 participants (40.4%), and the smallest group was aged between 51 and 60 and included 61 participants (12.4%). Regarding occupation, 216 participants (43.9%) were not working, 159 (32.3%) were employed, 38 (7.7%) were healthcare workers, and 79 (16.1%) were retired. In terms of the marital status, 202 participants (41.1%) were single, 262 (53.3%) were married, 21 (4.3%) were divorced, and seven (1.4%) were widowed. Educational levels varied among the participants, with the majority having attended college, 298 participants (60.6%). The duration of disease among the participants was predominantly over three years, accounting for 346 participants (70.3%). Regarding the number of medications used, 85 participants (17.3%) reported using no medications, and 133 (27.0%) used one medication. The demographic factors of the study participants are shown in Table 1.

**Table 1:** Demographic factors of the participants (N = 492)

		Count (N)	Column (%)
Gender	Male	163	33.1%
	Female	329	66.9%
Age	20-30	199	40.4%
	31-40	92	18.7%
	41-50	78	15.9%
	51-60	61	12.4%
	61 and more	62	12.6%
Occupation	Unemployed	216	43.9%
	Employee	159	32.3%
	Health-care workers	38	7.7%
	Retired‎	79	16.1%
Marital status	Single	202	41.1%
	Married	262	53.3%
	Divorced	21	4.3%
	Widow	7	1.4%
Educational level	Illiterate	4	0.8%
	Primary school	12	2.4%
	Intermediate school	15	3.0%
	High school	106	21.5%
	College	298	60.6%
	Higher education	57	11.6%
Duration of disease	1-3 months	26	5.3%
	3-6 months	18	3.7%
	6-12 months	28	5.7%
	1-3 years	74	15.0%
	>3 years	346	70.3%
Number of medications used	None	85	17.3%
	One medicine	133	27.0%
	Two medicines	115	23.4%
	Three medicines	60	12.2%
	4 medicines or more	99	20.1%

Prevalence of chronic conditions among the participants 

The most common condition reported was diabetes mellitus, affecting 115 participants (23.4%). Asthma was the second most prevalent, with 61 participants (12.4%), followed by hyperlipidemia (high cholesterol) with 43 participants (8.7%) and gastroesophageal reflux disease (GERD) with 40 participants (8.1%). Twenty-seven participants (5.5%) reported having multiple diseases, and 16 participants (3.3%) had other diseases.

Less common conditions included cardiovascular disease(2%), migraine (1.8%), eczema (1.2%), eye pathologies such as glaucoma and keratoconus (0.8%), polycystic ovary syndrome (PCOS) and gout, each affecting two participants (0.4%).

**Figure 1:**
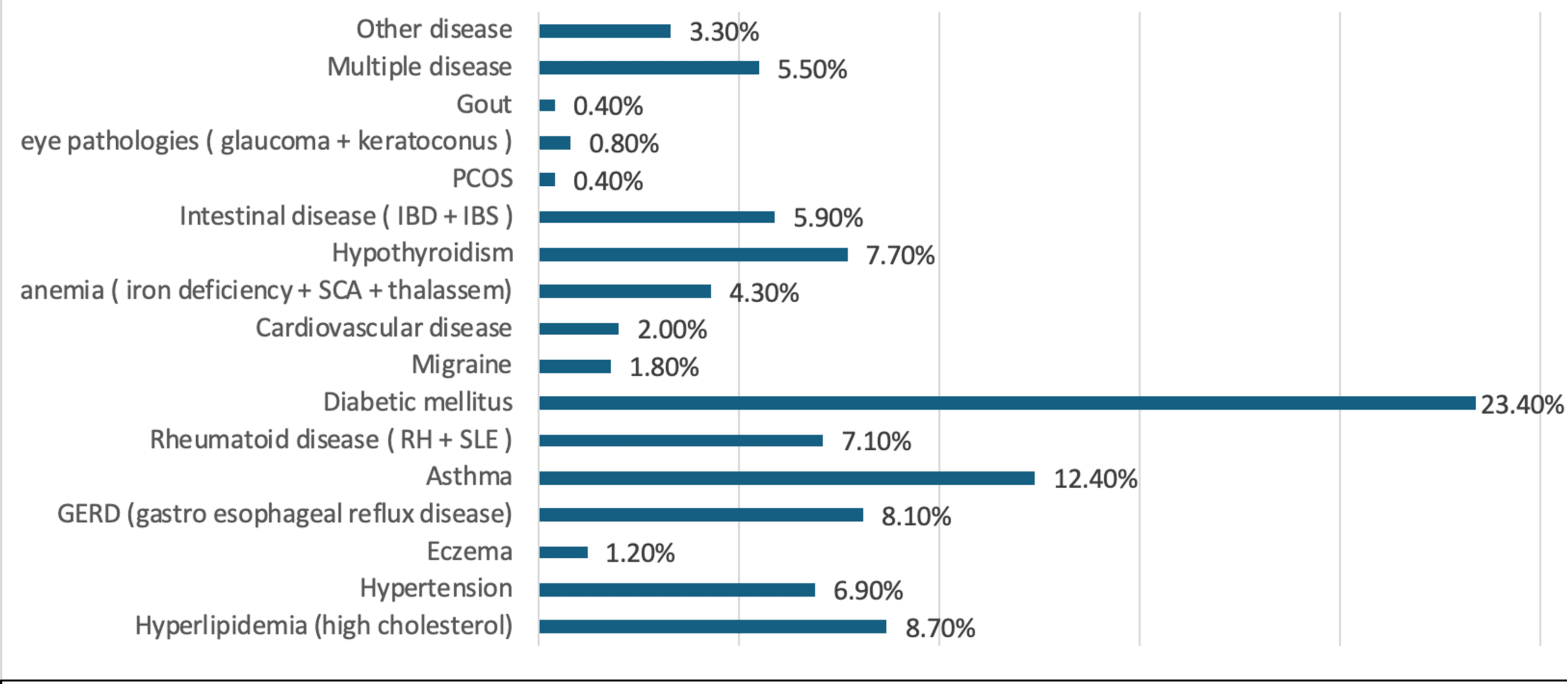
Prevalence of chronic conditions among the participants GERD: gastroesophageal reflux disease, RA: rheumatoid arthritis, SLE: systematic lupus erythematosus, IBD: irritable bowel disease, IBS: irritable bowel syndrome, PCOS: polycystic ovary syndrome, SCA: sickle cell anemia

Prevalence of medication adherence and depression among the participants

According to the Adherence to Refills and Medications Scale (ARMS) (Table 2), 191 participants (38.8%) were classified as not adherent, while 301 participants (61.2%) were adherent to their medication regimens. Depression severity, assessed using the PHQ-9, showed that 117 participants (23.8%) experienced none to minimal depression, 123 participants (25.0%) had mild depression, 100 participants (20.3%) had moderate depression, 81 participants (16.5%) had moderately severe depression, and 71 participants (14.4%) suffered from severe depression as determined by the PHQ-9 scores.

**Table 2:** Prevalence of adherence to medications and depression prevalence (N = 492) ARMS: Adherence to Refills and Medications Scale, PHQ-9: Patient Health Questionnaire-9

		Count (N)	Column (%)
Adherence to Refills And Medications Scale (ARMS)	Not adherent	191	38.8%
	Adherent	301	61.2%
PHQ-9 categories	None-minima	117	23.8%
	Mild depression	123	25.0%
	Moderate depression	100	20.3%
	Moderately severe depression	81	16.5%
	Severe depression	71	14.4%

Relationship between medication adherence and participants' demographic factors 

The ARMS was used to assess the relationship between adherence to medications and various demographic factors among the participants as outlined in Table 3. The analysis revealed significant differences in adherence rates based on gender, age, occupation, marital status, educational level, and number of medications used. In terms of gender, males demonstrated a higher adherence rate, with 117 (71.8%) adherent and 46 (28.2%) not adherent, compared to females, of whom 184 (55.9%) were adherent and 145 (44.1%) were not adherent (p = 0.001). Age also significantly influenced adherence, with older participants showing better adherence. Specifically, participants aged 61 or more had the highest adherence rate of 83.9% (52 adherent, 10 not adherent), while the 20-30 age group had the lowest adherence rate of 53.3% (106 adherent, 93 not adherent) (p = 0.001). Occupation also affected adherence, with retired individuals showing the highest adherence rate at 78.5% (62 adherent, 17 not adherent). Conversely, those unemployed had a lower adherence rate of 54.6% (118 adherent, 98 not adherent) (p = 0.003). Marital status revealed significant differences in adherence. Married participants had a higher adherence rate of 68.3% (179 adherent, 83 not adherent) compared to single participants, who had an adherence rate of 55.4% (112 adherent, 90 not adherent). Divorced participants had the lowest adherence rate at 28.6% (six adherent, 15 not adherent) (p < 0.001). Educational level showed a trend toward higher adherence with higher education, although this was not statistically significant (p = 0.064). Participants with higher education had the highest adherence rate at 77.2% (44 adherent, 13 not adherent), while illiterate participants had the lowest adherence rate at 25.0% (one adherent, three not adherent). The duration of the disease did not show a significant effect on adherence (p = 0.991). However, the number of medications used was significantly related to adherence (p < 0.001). Participants using four or more medications had the highest adherence rate at 72.7% (72 adherent, 27 not adherent), whereas those using one medication had an adherence rate of 61.7% (82 adherent, 51 not adherent).

**Table 3:** Relationship between adherence to medications and demographic factors Analysis was performed using the chi-square test and ANOVA test. * P-values less than 0.05 are deemed statistically significant. ARMS: Adherence to Refills and Medications Scale

		Adherence to Refills and Medications Scale (ARMS)				
		Not adherent		Adherent		P-value
		Count (N)	Row (%)	Count (N)	Row (%)	
Gender	Male	46	28.2%	117	71.8%	0.001*
	Female	145	44.1%	184	55.9%	
Age	20-30	93	46.7%	106	53.3%	0.001*
	31-40	35	38.0%	57	62.0%	
	41-50	30	38.5%	48	61.5%	
	51-60	23	37.7%	38	62.3%	
	61 or more	10	16.1%	52	83.9%	
Occupation	Unemployed	98	45.4%	118	54.6%	0.003*
	Employee	62	39.0%	97	61.0%	
	Health-care workers	14	36.8%	24	63.2%	
	Retired‎	17	21.5%	62	78.5%	
Marital status	Single	90	44.6%	112	55.4%	0.0001*
	Married	83	31.7%	179	68.3%	
	Divorced	15	71.4%	6	28.6%	
	Widow	3	42.9%	4	57.1%	
Educational level	Illiterate	3	75.0%	1	25.0%	0.064*
	Primary school	5	41.7%	7	58.3%	
	Intermediate school	7	46.7%	8	53.3%	
	High school	38	35.8%	68	64.2%	
	College	125	41.9%	173	58.1%	
	Higher education	13	22.8%	44	77.2%	
Duration of disease	1-3 months	11	42.3%	15	57.7%	0.991
	3-6 months	7	38.9%	11	61.1%	
	6-12 months	10	35.7%	18	64.3%	
	1-3 years	28	37.8%	46	62.2%	
	>3 years	135	39.0%	211	61.0%	
Number of medications used	One medicine	51	38.3%	82	61.7%	0.0002*
	Two medicines	42	36.5%	73	63.5%	
	Three medicines	20	33.3%	40	66.7%	
	4 medicines or more	27	27.3%	72	72.7%	

Relationship between depression severity and various demographic factors

Table 4 presents the relationship between depression severity, as measured by the PHQ-9, and various demographic factors. Gender, age, occupation, and marital status were all significantly related to depression severity (p < 0.05), while educational level, duration of disease, and number of medications used did not show significant associations. Regarding gender, males exhibited lower rates of depression compared to females. Among males, 62 (38.0%) had none to minimal depression, 44 (27.0%) had mild depression, 23 (14.1%) had moderate depression, 17 (10.4%) had moderately severe depression, and 17 (10.4%) had severe depression. By contrast, among females, 55 (16.7%) had none to minimal depression, 79 (24.0%) had mild depression, 77 (23.4%) had moderate depression, 64 (19.5%) had moderately severe depression, and 54 (16.4%) had severe depression (p < 0.001). Age also significantly influenced depression severity. Participants aged 61 or older had the highest proportion with no to minimal depression (34 participants, 54.8%) and the lowest with severe depression (three participants, 4.8%). Conversely, those aged 20-30 had the highest rates of severe depression (47 participants, 23.6%) and moderately severe depression (45 participants, 22.6%) (p < 0.001). Occupation was another significant factor. Retired participants had the highest rate of no to minimal depression (37 participants, 46.8%) and the lowest rates of moderate, moderately severe, and severe depression. Those unemployed had higher rates of depression across all categories, with 44 participants (20.4%) experiencing severe depression (p < 0.001). Marital status showed a significant relationship with depression severity. Married participants had the highest rate of none to minimal depression (83 participants, 31.7%) and the lowest rate of severe depression (17 participants, 6.5%). Single participants had higher rates of severe depression (47 participants, 23.3%) and moderately severe depression (45 participants, 22.3%) (p < 0.001). Educational level, although not statistically significant (p = 0.487), showed a trend where higher education was associated with lower depression severity. Participants with higher education had higher rates of none to minimal depression (17 participants, 29.8%) and lower rates of severe depression (seven participants, 12.3%). The duration of the disease did not show a significant association with depression severity (p = 0.435). However, a trend was observed where those with disease durations longer than three years had relatively stable rates across all categories of depression. Finally, the number of medications used also did not show a significant relationship with depression severity (p = 0.155). Participants using no medications had a higher proportion of severe depression (17 participants, 20.0%) compared to those using multiple medications, where severe depression rates were lower.

**Table 4:** Relationship between depression and demographic factors Analysis was performed using the chi-square test and ANOVA test. * P-values less than 0.05 are deemed statistically significant. PHQ-9: Patient Health Questionnaire-9

		PHQ-9 categories										
		None-minima		Mild depression		Moderate depression		Moderately severe depression		Severe depression		P-value
		Count (N)	Row (%)	Count (N)	Row (%)	Count (N)	Row (%)	Count (N)	Row (%)	Count (N)	Row (%)	
Gender	Male	62	38.0%	44	27.0%	23	14.1%	17	10.4%	17	10.4%	0.0001*
	Female	55	16.7%	79	24.0%	77	23.4%	64	19.5%	54	16.4%	
Age	20-30	32	16.1%	36	18.1%	39	19.6%	45	22.6%	47	23.6%	0.0001*
	31-40	16	17.4%	22	23.9%	23	25.0%	20	21.7%	11	12.0%	
	41-50	14	17.9%	24	30.8%	25	32.1%	9	11.5%	6	7.7%	
	51-60	21	34.4%	23	37.7%	9	14.8%	4	6.6%	4	6.6%	
	61 or more	34	54.8%	18	29.0%	4	6.5%	3	4.8%	3	4.8%	
Occupation	Unemployed	37	17.1%	48	22.2%	47	21.8%	40	18.5%	44	20.4%	0.0003*
	Employee	37	23.3%	42	26.4%	34	21.4%	26	16.4%	20	12.6%	
	Health-care workers	6	15.8%	9	23.7%	10	26.3%	9	23.7%	4	10.5%	
	Retired‎	37	46.8%	24	30.4%	9	11.4%	6	7.6%	3	3.8%	
Marital status	Single	29	14.4%	37	18.3%	44	21.8%	45	22.3%	47	23.3%	0.00004*
	Married	83	31.7%	78	29.8%	49	18.7%	35	13.4%	17	6.5%	
	Divorced	3	14.3%	7	33.3%	6	28.6%	0	0.0%	5	23.8%	
	Widow	2	28.6%	1	14.3%	1	14.3%	1	14.3%	2	28.6%	
Educational level	Illiterate	0	0.0%	1	25.0%	0	0.0%	2	50.0%	1	25.0%	0.487
	Primary school	4	33.3%	1	8.3%	4	33.3%	1	8.3%	2	16.7%	
	Intermediate school	4	26.7%	4	26.7%	1	6.7%	3	20.0%	3	20.0%	
	High school	24	22.6%	33	31.1%	21	19.8%	13	12.3%	15	14.2%	
	College	68	22.8%	66	22.1%	64	21.5%	57	19.1%	43	14.4%	
	Higher education	17	29.8%	18	31.6%	10	17.5%	5	8.8%	7	12.3%	
Duration of disease	1-3 months	6	23.1%	9	34.6%	2	7.7%	7	26.9%	2	7.7%	0.435
	3-6 months	2	11.1%	4	22.2%	4	22.2%	5	27.8%	3	16.7%	
	6-12 months	3	10.7%	11	39.3%	6	21.4%	3	10.7%	5	17.9%	
	1-3 years	20	27.0%	15	20.3%	14	18.9%	15	20.3%	10	13.5%	
	>3 years	86	24.9%	84	24.3%	74	21.4%	51	14.7%	51	14.7%	
Number of medications used	None	15	17.6%	26	30.6%	18	21.2%	9	10.6%	17	20.0%	0.155
	One medicine	39	29.3%	29	21.8%	20	15.0%	30	22.6%	15	11.3%	
	Two medicines	27	23.5%	24	20.9%	29	25.2%	19	16.5%	16	13.9%	
	Three medicines	10	16.7%	15	25.0%	17	28.3%	9	15.0%	9	15.0%	
	4 medicines or more	26	26.3%	29	29.3%	16	16.2%	14	14.1%	14	14.1%	

Relationship between the participants' chronic condition, medication adherence, and depression severity

Table 5 examines the relationship between the type of chronic condition, medication adherence, and depression severity among the participants. For medication adherence, significant differences were observed among different chronic conditions (p = 0.025). Participants with hyperlipidemia showed an adherence rate of 58.1% (25 adherent, 18 not adherent). Hypertension had one of the highest adherence rates at 79.4% (27 adherent, seven not adherent), while GERD patients were evenly split, with 50.0% adherent and 50.0% not adherent (20 participants each). Asthma patients had a notably lower adherence rate of 41.0% (25 adherent, 36 not adherent). For diabetes mellitus, the adherence rate was 68.7% (79 adherent, 36 not adherent), and hypothyroidism had an adherence rate of 76.3% (29 adherent, nine not adherent). Regarding depression severity, differences among various chronic conditions were not statistically significant (p = 0.121). However, notable trends were observed.

Hypertension patients showed lower depression levels, with 35.3% (12 participants) having none to minimal depression and 5.9% (two participants) experiencing severe depression. GERD patients had a higher proportion of severe depression at 15.0% (six participants). Asthma patients exhibited high levels of severe depression at 24.6% (15 participants) and moderately severe depression at 13.1% (eight participants). By contrast, those with rheumatoid disease had lower rates of none to minimal depression at 8.6% (three participants) but higher rates of moderately severe and severe depression at 28.6% each (10 participants). Diabetes mellitus patients comprised 27.0% (31 participants) with none to minimal depression and 10.4% (12 participants) with severe depression. Participants with intestinal diseases had low rates of none to minimal depression at 6.9% (two participants) but higher rates of severe depression at 20.7% (six participants). Patients with multiple diseases showed diverse depression levels, with 33.3% (nine participants) having none to minimal depression and 14.8% (four participants) having severe depression. Those with "other diseases" had 37.5% (six participants) with none to minimal depression and 18.8% (three participants) with severe depression.

**Table 5:** Relationship between the type of chronic condition and depression and adherence to medication Analysis was performed using the chi-square test. * P-values less than 0.05 are deemed statistically significant. ARMS: Adherence to Refills and Medications Scale, PHQ-9: Patient Health Questionnaire-9, GERD: gastroesophageal reflux disease, RA: rheumatoid arthritis, SLE: systematic lupus erythematosus, IBD: irritable bowel disease, IBS: irritable bowel syndrome, PCOS: polycystic ovary syndrome

		Adherence to Refills and Medications Scale (ARMS)				PHQ-9 categories									
		Not adherent		Adherent		None-minima		Mild depression		Moderate depression		Moderately severe depression		Severe depression	
		Count (N)	Row (%)	Count (N)	Row (%)	Count (N)	Row (%)	Count (N)	Row (%)	Count (N)	Row (%)	Count (N)	Row (%)	Count (N)	Row (%)
Chronic condition	Hyperlipidemia (high cholesterol)	18	41.9%	25	58.1%	13	30.2%	15	34.9%	6	14.0%	6	14.0%	3	7.0%
	Hypertension	7	20.6%	27	79.4%	12	35.3%	10	29.4%	6	17.6%	4	11.8%	2	5.9%
	Eczema	2	33.3%	4	66.7%	2	33.3%	2	33.3%	2	33.3%	0	0.0%	0	0.0%
	GERD	20	50.0%	20	50.0%	6	15.0%	10	25.0%	9	22.5%	9	22.5%	6	15.0%
	Asthma	36	59.0%	25	41.0%	14	23.0%	11	18.0%	13	21.3%	8	13.1%	15	24.6%
	Rheumatoid disease (RA + SLE)	13	37.1%	22	62.9%	3	8.6%	4	11.4%	10	28.6%	10	28.6%	8	22.9%
	Diabetic mellitus	36	31.3%	79	68.7%	31	27.0%	37	32.2%	20	17.4%	15	13.0%	12	10.4%
	Migraine	4	44.4%	5	55.6%	2	22.2%	2	22.2%	4	44.4%	1	11.1%	0	0.0%
	Cardiovascular disease	4	40.0%	6	60.0%	3	30.0%	3	30.0%	2	20.0%	1	10.0%	1	10.0%
	Anemia (iron deficiency + thalassemia)	10	47.6%	11	52.4%	2	9.5%	3	14.3%	6	28.6%	4	19.0%	6	28.6%
	Hypothyroidism	9	23.7%	29	76.3%	9	23.7%	10	26.3%	6	15.8%	10	26.3%	3	7.9%
	Intestinal disease (IBD + IBS)	11	37.9%	18	62.1%	2	6.9%	9	31.0%	5	17.2%	7	24.1%	6	20.7%
	PCOS	2	100.0%	0	0.0%	1	50.0%	0	0.0%	0	0.0%	0	0.0%	1	50.0%
	Eye pathologies (glaucoma + keratoconus)	2	50.0%	2	50.0%	1	25.0%	1	25.0%	0	0.0%	2	50.0%	0	0.0%
	Gout	1	50.0%	1	50.0%	1	50.0%	0	0.0%	0	0.0%	0	0.0%	1	50.0%
	Multiple disease	9	33.3%	18	66.7%	9	33.3%	4	14.8%	8	29.6%	2	7.4%	4	14.8%
	Other diseases	7	43.8%	9	56.3%	6	37.5%	2	12.5%	3	18.8%	2	12.5%	3	18.8%
P-value		0.025				0.121									

Relationship between depression severity and medication adherence

The association between depression severity, as measured by the PHQ-9 categories, and adherence to medications among the participants is illustrated in Table 6. Medication adherence significantly varied with depression severity (p < 0.001). Among participants with no minimal depression, 82.1% (96 participants) were adherent to their medications, serving as the reference group. Those with mild depression had a reduced adherence rate of 64.2% (79 adherent, 44 not adherent), with a relative risk (RR) of 2.546 (95% confidence interval (CI): 1.39-4.64), indicating that they were over two and a half times more likely to be non-adherent compared to those with none to minimal depression (p = 0.002). Participants with moderate depression showed an adherence rate of 56.0% (56 adherent, 44 not adherent), with an RR of 3.59 (95% CI: 1.94-6.65), making them more than three and a half times as likely to be non-adherent (p < 0.001). Similarly, those with moderately severe depression had an adherence rate of 55.6% (45 adherent, 36 not adherent), with an RR of 3.66 (95% CI: 1.92-6.96), also more than three and a half times more likely to be non-adherent (p < 0.001). The most pronounced effect was seen in participants with severe depression, who had the lowest adherence rate at 35.2% (25 adherent, 46 not adherent). The RR for severe depression was 8.41 (95% CI: 4.26-16.57), indicating that they were more than eight times more likely to be non-adherent compared to those with none to minimal depression (p < 0.001).

**Table 6:** Relationship between depression and adherence to medications Analysis was performed using the chi-square test. * P-values less than 0.05 are deemed statistically significant. ARMS: Adherence to Refills and Medications Scale, PHQ-9: Patient Health Questionnaire-9, RR: relative risk, CI: confidence interval

		Adherence to Refills and Medications Scale (ARMS)					
		Not adherent		Adherent			
		Count (N)	Row (%)	Count (N)	Row (%)	RR (95% CI)	P-value
PHQ-9 categories	None-minima	21	17.9%	96	82.1%	Reference	
	Mild depression	44	35.8%	79	64.2%	2.546 (1.39-4.64)	0.002*
	Moderate depression	44	44.0%	56	56.0%	3.59 (1.94-6.65)	0.0001*
	Moderately severe depression	36	44.4%	45	55.6%	3.66 (1.92-6.96)	0.0002*
	Severe depression	46	64.8%	25	35.2%	8.41 (4.26-16.57)	0.0001*

## Discussion

The findings of this cross-sectional study provide significant insights into the relationship between demographic factors, chronic conditions, medication adherence, and depression among patients with chronic diseases in the Middle East. The data reveal complex interactions that have important implications for healthcare providers and policymakers aiming to improve patient outcomes in this population.

Demographic factors and medication adherence

In the current study, we found that the prevalence of adherence of patients with chronic conditions to their medications was 61.2%. In a previous study conducted in Tabuk, the authors reported a slightly higher rate of adherence of 76.44% [12], 77.1% among patients with chronic conditions in Jeddah, Saudi Arabia [13], and 66% among patients with diabetic mellitus in Jazan, Saudi Arabia [14]. The study demonstrated that demographic factors such as gender, age, occupation, and marital status significantly influence medication adherence. Female participants were more likely to be non-adherent compared to males (p = 0.001). This finding aligns with previous research suggesting that women often face greater barriers to medication adherence leading to lower adherence rates to their medications [14,15], possibly due to higher caregiving responsibilities and economic challenges and more worries about adverse effects of the medications [16].

Age also played a crucial role, with younger participants (20-30 years old) showing the highest rates of non-adherence (46.7%), while those aged 61 and older had the highest adherence rates (83.9%) (p = 0.001). This trend is consistent with the literature indicating that older adults often have better adherence due to greater health awareness and routine management of chronic conditions [17,18]. However, other studies showed that younger age is associated with better adherence due to increased forgetfulness among older patients and multi-pharmacy among older patients [19] and others did not find a significant difference between different ages [20-22].

Occupational status influenced adherence, with non-working individuals showing higher non-adherence rates (45.4%) compared to retired individuals who exhibited the highest adherence rates (78.5%) (p = 0.003). Employment can impact adherence through factors like time constraints and stress, whereas retired individuals might have more time to focus on their health. However, several studies reported no significant differences in adherence according to occupation [14,19].

Marital status was another significant factor, with single participants showing higher non-adherence rates (44.6%) compared to married participants (31.7%) (p < 0.001). Being married often provides emotional and logistical support that can enhance adherence [23,24].

Chronic conditions and medication adherence

The study found that the type of chronic condition significantly affects medication adherence (p = 0.025). For instance, participants with hypertension had the highest adherence rates (79.4%), whereas those with asthma showed lower adherence (41.0%). This disparity can be attributed to the perceived severity and manageability of the conditions. Hypertension, often managed with a single daily medication, might be perceived as less intrusive than asthma, which requires more complex management strategies like inhalers and frequent monitoring. Diabetes mellitus patients also demonstrated relatively high adherence (68.7%), likely due to the well-established education and support systems for diabetes management.

Depression and its impact on medication adherence

A significant finding of this study is the strong inverse relationship between depression severity and medication adherence (p < 0.001). Participants with severe depression were more than eight times more likely to be non-adherent compared to those with none to minimal depression (RR = 8.41, 95% CI: 4.26-16.57). This finding is consistent with previous studies that highlight how depression can negatively affect cognitive functions, motivation, and the ability to follow treatment regimens [25,26]. In a previous study conducted by Eze-Nliam et al. (2010), the authors revealed a robust negative association: depression significantly hinders adherence to antihypertensive medications [27]. In addition, a previous meta-analysis showed that depressed patients had a 1.76 times risk of being non-adherent to their medications [8].

The graded increase in non-adherence with higher levels of depression severity suggests a need for integrated care approaches that simultaneously address mental health and chronic disease management. Mild to moderate depression was associated with more than double to triple the risk of non-adherence, emphasizing the necessity for early intervention in depressive symptoms to prevent deterioration in medication adherence [28,29].

Depression and demographic factors

Depression severity, assessed using the PHQ-9, showed that 25.0% of the patients had mild depression, 20.3% had moderate depression, 16.5% had moderately severe depression, and 14.4% suffered from severe depression. Similar results were reported in the literature showing that depression is common among patients with chronic conditions [30,31].

Depression was found to vary significantly with demographic factors. Females exhibited higher rates of severe depression compared to males (16.4% vs. 10.4%, p < 0.001) which was also reported in previous studies showing that women had a higher risk of developing depression [32,33]. Younger age groups (20-30 years) had the highest prevalence of severe depression (23.6%), whereas older participants (61 and older) had lower rates of severe depression (4.8%), which was also reported by a study by Patel R et al. [34]. These patterns underscore the need for targeted mental health interventions that consider age and gender differences.

Occupation also influenced depression levels, with non-working participants showing higher rates of severe depression (20.4%). This may reflect the psychological impact of unemployment and financial instability [35]. Marital status was another critical factor, with single participants exhibiting higher rates of severe depression (23.3%) compared to married participants (6.5%). The support and companionship provided by marriage could play a protective role against depression [36].

Implications for healthcare practice

These findings have multiple consequences for healthcare procedures. First and foremost, it is important to regularly screen for depression in individuals with chronic conditions, especially in those who have identifiable risk factors like being young, female, single, or unemployed. Combining mental health services with chronic disease management in integrated care models may enhance mental health and medication adherence. Healthcare professionals need to recognize the unique difficulties related to various chronic diseases and adjust their adherence approaches accordingly. Patients with asthma may receive added value from increased education and support in relation to their treatment plans. Likewise, focused efforts should be made to enhance adherence in individuals with low adherence rates, like younger people and those lacking social support.

Moreover, efforts in public health should target reducing the negative perceptions linked to mental health problems in the Middle East, promoting help-seeking behavior and adherence to treatment plans among individuals. Community-driven initiatives that offer social backing and information on how to manage chronic diseases and mental health issues may also prove to be advantageous.

Although important insights into the rates of depression and medication adherence among patients with chronic diseases in the Middle East are provided by this cross-sectional study, it is important to note several limitations. The use of an online survey shared on social media could lead to selection bias by excluding people without internet access or not active on these platforms. This may lead to certain demographic groups being underrepresented, which could potentially bias the results. Furthermore, using self-reported data can lead to response bias, as participants may give answers that are socially acceptable or remember information inaccurately. In addition, the cross-sectional design of the study prevents determining causality or evaluating the temporal connections between depression and medication adherence. Long-term research is needed to clarify the pattern of these connections as time progresses. Moreover, the size of the participants in the study, although suitable for the statistical methods selected, could constrain the ability to apply the findings to a wider group of patients with chronic illnesses in the Middle East.

Limitations

As an online questionnaire was used to collect data, the results may not represent the whole population of the Middle East. Furthermore, the participants were recruited via convenience sampling, which increases the possibility of selection bias. Moreover, this study had an unequal distribution between genders, age groups, and regions, which may impact the generalizability of the results.

## Conclusions

This study highlights that depression is a significant factor affecting the level of adherence among patients with chronic conditions. Furthermore, certain demographic factors such as gender, age, occupational status, and marital status significantly influence both adherence and depression levels. Addressing these issues requires a multifaceted approach that includes mental health support, tailored adherence strategies, and targeted public health interventions. By understanding and addressing the factors that influence medication adherence and depression, healthcare providers can improve outcomes for patients with chronic diseases.

## Appendices

Study questionnaire

Do you have chronic disease ? 

Yes 

No 

Gender: 

Male 

Female 

If you are female please choose one of the following: Pregnant 

Not pregnant 

Age: 

20-30 

31-40 

41-50 

51-60 

61 or more 

Occupation: 

Employed 

Unemployed 

Health-care worker 

Retired 

Marital status: 

Single 

Married 

Divorced 

Widow 

Educational level: 

Illiterate 

Primary school 

Intermediate school 

High school 

College 

Higher education 

Chronic disease: 

Diabetes mellitus 

Hypertension 

Hyperlipidemia (high cholesterol) 

Cardiovascular disease

Hypothyroidism 

GERD (gastroesophageal reflux disease) 

Rheumatoid disease 

Asthma 

Other: ……. 

Duration of disease: 

1-3 months 

3-6 months 

6-12 months 

1-3 years 

>3 years 

Number of medications used: 

None 

One medicine 

Two medicines 

Three medicines 

4 medicines or more 

Monthly income: 

Less than 10K 

From 10 to 15 K 

From 15 to 20 K 

From 20 to 30 K 

30 K or more 

Adherence to Refills and Medications Scale (ARMS)

I will now ask you how often you actually miss taking your medicines. There are no right or wrong answers. For each question, please answer “none of the time,” “some of the time,” “most of the time,” or “all of the time.”

**Table 7:** Adherence to Refills and Medications Scale (ARMS)

	None of the time	Some of the time	Most of the time	All of the time
1. How often do you forget to take your medicine?	0	1	2	3
2. How often do you decide not to take your medicine?	0	1	2	3
3. How often do you forget to get prescriptions filled?	0	1	2	3
4. How often do you run out of medicine?	0	1	2	3
5. How often do you skip a dose of your medicine before you go to the doctor?	0	1	2	3
6. How often do you miss taking your medicine when you feel better?	0	1	2	3
7. How often do you miss taking your medicine when you feel sick?	0	1	2	3
8. How often do you miss taking your medicine when you are careless?		1	2	3
9. How often do you change the dose of your medicines to suit your needs (like when you take more or less pill than you’re supposed to)?	0	1	2	3
10. How often do you forget to take your medicine when you are supposed to take it more than once a day?	0	1	2	3
11. How often do you put off refilling your medicines because they cost too much money?	0			3
12. How often do you plan ahead and refill your medicines before they run out?	0	1	2	3

PHQ-9 Scale

**Table 8:** Patient Health Questionnaire (PHQ-9)

Over the last 2 weeks, how often have you been bothered by any of the following problems?	Not at all	Several days	More than half the days	Nearly every day
1. Little interest or pleasure in doing things	0	1	2	3
2. Feeling down, depressed, or hopeless	0	1	2	3
3. Trouble falling or staying asleep, or sleeping too much	0	1	2	3
4. Feeling tired or having little energy	0	1	2	3
5. Poor appetite or overeating	0	1	2	3
6. Feeling bad about yourself – or that you are a failure or have let yourself or your family down	0	1	2	3
7. Trouble concentrating on things, such as reading the newspaper or watching television	0	1	2	3
8. Moving or speaking so slowly that other people could have noticed. Or the opposite – being so fidgety or restless that you have been moving around a lot more than usual	0	1	2	3
9. Thoughts that you would be better off dead, or of hurting yourself in some way	0	1	2	3
10. If you checked off any problems on this questionnaire so far, how difficult have these problems made it for you to do your work, take care of things at home, or get along with other people?	0	1	2	3
